# Under-representation of developing countries in the research literature: ethical issues arising from a survey of five leading medical journals

**DOI:** 10.1186/1472-6939-5-5

**Published:** 2004-10-04

**Authors:** Athula Sumathipala, Sisira Siribaddana, Vikram Patel

**Affiliations:** 1 Section of Epidemiology, Institute of Psychiatry, Kings College, London SE5 8AF UK; 2 Sri Jayewardenepura Postgraduate Teaching Hospital, Thalapathpitiya, Nugegoda, 10250, Sri Lanka; 3 London School of Hygiene & Tropical Medicine, London, UK; 4 Forum for Research and Development in Sri Lanka

## Abstract

**Background:**

It is widely acknowledged that there is a global divide on health care and health research known as the 10/90 divide.

**Methods:**

A retrospective survey of articles published in the BMJ, Lancet, NEJM, Annals of Internal Medicine & JAMA in a calendar year to examine the contribution of the developing world to medical literature. We categorized countries into four regions: UK, USA, Other Euro-American countries (OEAC) and (RoW). OEAC were European countries other than the UK but including Australia, New Zealand and Canada. RoW comprised all other countries.

**Results:**

The average contribution of the RoW to the research literature in the five journals was 6.5%. In the two British journals 7.6% of the articles were from the RoW; in the three American journals 4.8% of articles were from RoW. The highest proportion of papers from the RoW was in the Lancet (12%). An analysis of the authorship of 151 articles from RoW showed that 104 (68.9%) involved authorship with developed countries in Europe or North America. There were 15 original papers in these journals with data from RoW but without any authors from RoW.

**Conclusions:**

There is a marked under-representation of countries in high-impact general medical journals. The ethical implications of this inequity and ways of reducing it are discussed.

## Background

National and international bodies concerned with research ethics need to confront the greatest ethical challenge-the enormous inequities in global health research [1]. Thus, less than 10% of the world's research resources are earmarked for 90% of the health problems [2]. Though 93% of the world's burden of preventable mortality occurs in developing countries, too little research funding is targeted to health problems in those countries [3]. This divide in research funding is mirrored by concerns regarding a divide in the proportion of publications arising from medical research in developing countries. A recent survey of six leading psychiatric journals revealed that only 6% of the articles originated from, or described data arising from, regions of the world that accounted for over 90% of the global population [4]. Following this paper, the issue of under-representation of developing countries was debated and discussed in many journals and by journal editors [5-7].

The aim of this study is to investigate the publication bias beyond the field of psychiatry to determine the overall contribution of different regions of the world to the medical literature published in high-impact journals and, in particular, to quantify the developing world's contribution.

## Methods

The method used is the same as that which was used by two of the authors (AS and VP) in a recent survey of the international representation in the psychiatric literature [4]. A retrospective survey was conducted of all issues in one calendar year (the most recent, complete set available in the medical library in Colombo where data collection took place) of the following journals: BMJ, Lancet, NEJM, Annals of Internal Medicine & JAMA. These journals were selected because they have the highest impact amongst general medical journals [8]. These journals lay claim to their global legitimacy for many reasons: frequent publication, high impact, long history, credibility of the publisher, large numbers of full time editorial staff, membership of the International Committee of Medical Journal Editors, and influential joint statements [9]. The year for which data was collected was 2000 for all journals, except JAMA for which it was 1999 (all issues in year 2000 were not available). All articles, excluding the specified ones in each journal were reviewed. In the BMJ we excluded obituaries, multi-media, personal views, Minerva, news and soundings. In the Lancet we excluded Dissecting Room and news. In the Annals, we excluded on Being a doctor, Current clinical issues, Medical writings, Book notes, Ad Libitum and persona. In the NEJM, we excluded book reviews, This week in the journal and Abstracts. In JAMA, we excluded Medical news and Perspectives, Peace of my mind, JAMA hundred years ago, Abstracts, FDA, CDC, poetry and medicines, Books journals, New media, World in medicine and the section titled "from the JAMA websites". In the Lancet, editorials and commentaries were pooled together, because the commentaries in the Lancet resemble editorials in the other journals in terms of their contents. None of the other four journals had commentaries section.

For the allocation of regions of the world, we categorized countries into four regions: UK, USA, OEAC (Other Euro-American countries) and RoW (Rest of the World). OEAC were European countries other than the UK but including Australia, New Zealand and Canada. RoW comprised all other countries including Eastern Europe, Central and South America, Asia and Africa. We scrutinized each article to examine the country of the authors' affiliation address with a view to categorizing them into relevant regions of the world. All authors were noted including multiple authors in large multi-center studies. We also scrutinized the methods section of all the original articles to ascertain the origin of data with special emphasis on whether the research was carried out in RoW countries. The affiliation of the first, last and corresponding authors was also noted. We worked with a negative bias against the USA and the UK from where the journals originated. Therefore articles, which included authors from the RoW, were considered as arising from the RoW even if the first author is from the UK, USA or OEAC. Similarly, articles from the OEAC, which involved collaborations from the UK or USA, were included in the OEAC category. In the event of a collaborative study between the USA and the UK, the allocation to region was based on the institutional affiliation of the first author. The RoW category included collaborative studies between any country in the RoW and developed nations.

In some cases the origin of the author was difficult, particularly when there were two places named as their attached institutions. For an example when one was in USA while the other is in Kenya. We used our best guess in these instances. For an example the two authors AS and VP in this paper were based in RoW at the time we did this research but were employed in UK but with strong affiliations to the RoW countries where they were born and did their research. Attempts to analyse the nationality of the authors was therefore abandoned.

## Results

The contribution of the RoW to the research literature surveyed in these five high-impact journals was 6.5%. In the two British journals, 7.6% of the articles were from the RoW, whilst the proportion in the three American journals was 4.8%. This averages hides the fact that there is considerable variation between journals; thus, around 3.5% of articles in the two journals of national medical associations of the UK (BMJ) and USA (JAMA) were from the RoW as compared to 12% in the Lancet articles (Table 1). Indeed, more than half the articles from the RoW were published in just one journal, the Lancet (Table 2).

**Table 1:** The contribution of regions to the research literature in five leading journals

	BMJ		Lancet		NEJM		JAMA		ANNALS		Total	
Editorials	261		350^4^		124		102		39		876	
UK	160	61.4%	167	47.7%	10	8.0%	02	1.9%	01	2.4%	340	38.8%
USA	38	14.6%	87	24.9%	97	78.3%	97	95.0%	35	90.0%	354	40.4%
OEAC^1^	56	21.4%	89	25.4%	14	11.3%	02	1.9%	03	7.6%	164	18.7%
RoW^2^	07	2.6%	07	2.0 %	03	2.4%	01	0.9%	00	0.0%	18	2.6%
Original papers	322		307		218		227		115		1189	
UK	216	67.0%	66	21.5 %	06	2.7%	04	1.8%	03	2.6%	295	24.8%
USA	22	7.0%	38	12.4%	107	49.1%	173	76.2%	81	70.4%	421	35.4%
OEAC	76	23.6%	136	44.3 %	78	35.8%	42	18.5%	23	20.0%	355	29.9%
RoW	08	2.4%	67	21.8%	27	12.4%	08	3.5%	08	7.0%	118	9.9%
Correspondence	1118		1043^5^		894		605		251		3911	
UK	829	74%	374	35.8%	30	3.4%	13	2.1%	04	1.6%	1250	32%
USA	69	6%	141	13.5%	601	67.2%	506	83.6%	183	72.9%	1500	38.4%
OEAC	174	16%	400	38.3%	212	23.7%	71	11.7%	49	19.5%	906	23.2%
RoW	46	4%	128	12.2%	51	5.7%	15	2.5%	15	6.0%	255	6.5%
Review articles	286		50		65		36		66		503	
UK	195	68.2%	17	34%	04	6.2%	01	2.7%	01	1.6%	218	43.3%
USA	29	10.2%	15	30%	48	73.8%	28	78.0%	61	92.4%	181	36%
OEAC	54	18.8%	14	28%	13	20.0%	04	11.1%	04	6.0%	89	17.7%
RoW	08	2.8%	04	08%	00	0.0%	03	8.2%	00	0.0%	15	3%
Others^3^	47		208		149		145		27		576	
UK	31	66.0%	68	32.8%	03	02.0%	00	00.0%	00	00.0%	102	17.7%
USA	08	17.0%	34	16.3%	118	79.3%	120	82.7%	25	92.6%	305	53%
OEAC	04	8.5%	78	37.5%	22	14.7 %	16	11.0%	01	3.7%	121	21%
RoW	04	8.5%	28	13.4%	06	4.0%	09	6.2%	01	3.7%	48	8.3%
Global total	2034		1958		1450		1115		498		7055	
RoW total	73	3.6%	234	12%	87	6%	36	3.2%	24	4.8%	454	6.4 %

1. OEAC (Other Euro-American countries), OEAC were European countries other than the UK, Australia, New Zealand and Canada.

2. RoW comprised all other countries (i.e. all countries in Eastern Europe, Central and South America, Asia and Africa)

3. Others include: for **The Lancet**: case reports, view points, public letters, essays, public health, violence in health, department of medical history, the world, world ideas, health and human rights, development of ethics, eponymous, clinical picture, reportage, adverse drug reactions, personal papers, medicine and law, personal papers; for the **JAMA**: Patients relationships, grand rounds, clinical cross roads, rational clinical examinations, medicine and media, commentary, updates, letters from counters, public opinion and health, medical literature, health law and ethics, clinical cross roads; for the **NEJM**: images, clinical problem solving, case records from Massachusetts general hospital, clinical implications of basic research, sounding board, clinical problem solving, special articles, for the **BMJ**: drug point, practice and results, quality of life, history, practice point, lessons of every week; for the **Annals**: past present and future, time and medicine, social means of medicine, technology of time, personal time, media and publication, NIH conference, in the balance, abroad.

4. The Lancet had 52 editorials and 298 commentaries. Editorials were written in house.

5. The Lancet had 262 research letters. We did not include these either in the correspondence or with the Original papers category. Other journals did not have such a category to compare.

**Table 2:** Proportion of articles contributed by different regions of the RoW category to the total number of editorials, original articles and reviews.

	**BMJ**	**Lancet**	**NEJM**	**JAMA**	**Annals**	**Total**^1^**(%)**
India	2	1	2	0	0	05 (3)
China	2	5	6	1	1	15 (10)
Other Asian	6	8	3	2	0	19 (12)
Sub Saharan Africa	8	25	2	1	0	36 (24)
Latin America	2	11	4	5	2	24 (16)
Middle East/North Africa	0	6	1	0	0	07 (5)
Japan	0	8	5	0	4	17 (11)
Israel	3	6	4	1	0	14 (9)
Multi national	0	8	3	2	1	15 (10)
**Total**^2 ^(%)	23 (15)	78 (52)	30 (20)	12 (8)	8 (5)	151

1 The % figures in this column represent the contribution of the specific country or region to the RoW papers in the table

2 The % figures in this row represent the contribution of the specific journal to the total RoW papers in the table.

Note: This analysis only includes Original Articles, Editorials and Review Papers

Within journals, variations in regional contributions from other regions were also notable. Thus, the proportion of articles from the UK was highest in the two journals published in the UK (BMJ and Lancet) while the proportion of articles from the USA was highest in the three journals published in the USA. Table 2 shows the relative contribution of different regions of the RoW category to the total number of editorials, original articles and reviews. Two developed countries (Japan and Israel) contribute a fifth of the literature from RoW, while the two most populous countries in the world (India and China) contribute about 13% together. A detailed analysis of the authorship of the 151 RoW articles showed that 104 (68.9%) involved collaborative authorship with developed countries in Europe or North America; only 43 (31.1%) were entirely independent efforts from the RoW.

There were 118 original papers with at least one author from the RoW. Forty-five (38%) of them had a first author from the RoW; 32 (27%) had a last author from the RoW; and 36 (30%) had a corresponding author from the RoW. Only 25 (21%) of them had first, last and corresponding authors all from the RoW. There were 15 original papers in these journals with data from the RoW but without any authors from the RoW; of these 15, nine were in Lancet, four in BMJ and one each in JAMA and NEJM. Thus, the majority of the original articles originating from the RoW had contribution from the developed world authors.

## Discussion

This study presents findings of a survey of articles published in five high-impact general medical journals with the objective of describing the developing world's contribution to research literature over a single calendar year.

Our way of classifying countries into developed (UK, USA, OEAC) and developing countries (RoW) has obvious inadequacies but we followed the same method adopted in an earlier paper [4]. Other euro-American countries (OEAC) shared many cultural and economic features. Rest of the world (RoW) included Eastern Europe, which although culturally related to Western Europe was not economically on the same level, and Japan, which was highly developed economically but did not share many cultural factors with OEAC.

The key finding of our survey is that, only 6.5% of the publications in these journals have authors from countries where 90% of the world's population lives. There is a severe under representation of biomedical research from a vast section of the world in these five journals. In addition, there are a small but significant number of articles with data from the developing world without a single author from these countries. This is a troublesome finding that some would refer as 'safari research'. It is a separate ethical issue and journal editors need to look at it carefully.

The reasons for under representation of researchers based in developing countries may include research barriers such as lack of funding, poor facilities, limited technical support and inadequate training. Many researchers from developing countries do not speak English as their first language. Fear of rejection by the journals, uncertainty about which journal would be best to publish research, a lack of the culture of publication, competing clinical commitments, different ministry and donor driven agendas for research, are some of the unseen barriers facing developing country researchers (10). The editors of leading medical journals may not have paid sufficient attention to these barriers, real and perceived, that clinical researcher in developing countries face [11].

We acknowledge that many developing country researchers choose to publish their work in national or regional journals that are not as high-impact as the journals we have reviewed. However, many researchers, irrespective of the country of their origin, also prefer to publish their work in journals with a high impact factor and circulation. This, inevitably, leads to a focus on leading journals published from the UK or USA. However, journals may be under some pressure to publish material, which is relevant to the majority of their readership. Thus, it is not surprising that journals from the UK have the majority of articles from UK institutions and similarly, journals from the USA tend to have most of their articles from US institutions. The BMJ admits that although the BMJ aspires to be more international, they cannot forget that they reach 80% doctors in Britain [12]. However all these journals are financially highly successful in global markets. Their international success brings responsibilities to the global community they serve and profits from [11].

Our findings are in agreement with other published findings in this area [13]. In a study of randomised controlled trials published in leading medical journals many of the diseases afflicting the south are understudied [14]. Even in the field of tropical medicine there were few contributions originating from countries with a low human development index (HDI) [15]. Another study, where the number of biomedical articles were normalized to the number of publications per million population, also shows an under representation of Asia, Africa and South America. The authors demonstrated that the number of biomedical publications increase according to the economic ranking of the country [16]. The same authors have shown in a similar study that publications per million population are more closely related to gross national product (GNP) and research and development expenditure [17]. The inescapable conclusion of this research is that USA, UK and other European countries dominate biomedical research, in part because of their wealth and investment in research. Irrespective of the method used to categorize countries in developing world, (by GNP, HDI or any other method), generation of new knowledge in biomedical research in these nations is insufficient. In a recent Nature paper on science publications Japan occupies 4th place, Israel 15th, China 19th, and India 22nd in the rank order of nations based on their share of top 1% of highly cited publications. Unsurprisingly the USA and UK occupies the two top positions [18].

There are many reasons to strive for a more just international distribution of biomedical research in leading journals. The burden of disease in the world falls heavily on developing countries and the pattern of this burden is likely to change in the next twenty years. Diseases like TB, HIV, Malaria, Dengue, and viral hemorrhagic fevers are now no longer tropical with increase in international travel, global warming, refugees, economic and military intervention and conflicts. Newly emerging infections from tropics such as SARS and bird flu can have devastating effects far away from tropics. In addition, diseases such as diabetes and obesity can no longer be considered as diseases in the developed world. As the impact and burden of these diseases are more in developing countries more research publications are needed from South.

Clinicians and policy makers in many developing countries have limited access to international journals because medical libraries often need to make a choice from a number of journals due to financial constraints. Often, it is the high-impact journal, which is subscribed to. Thus, the proportion of international representation in these leading journals may be a crucial factor in influencing health policies in many countries. The importance of research goes well beyond its impact on health policies [11]. Thus, research on the epidemiology and management of diseases in different health systems raises the probability of identifying risk factors for diseases and identifying innovative approaches to their management. This fact was well reflected in the multi-center eclampsia trial [19]. Until this trial was carried out in Africa, South America and India, the subject of using magnesium sulfate in the management of eclampsia was controversial. The other good example is the only meningitis B vaccine in the world, which was developed by the Carlos J. Finlay Institute in Cuba and now saves lives all over the world [20].

Providing free journals electronically to the developing countries is a commendable step in correcting the thirst for new information [21]. However the one-way flow of information by making journals electronically free to developing world is unlikely to be sufficient [22]. By providing journals free to 'RoW', and propagating research which has been conducted in countries where only 10% of the disease burden is experienced is itself a moral and ethical issue.

Ultimately, we believe that strengthening the health research capacity in developing world and providing reasonable opportunities for publications arising from the developing world are critical, not only for achieving biomedical research publication equity, but also advancing medical science.

## Competing interests

We would like to state here that our conflict of interest if at all would be due to the fact that we come from the developing world.

## Authors' contributions

All authors were involved in all the components of this paper, right from the start, in formulating and developing the idea, collecting the data, drafting the first version and revising it and approving the final version.

## Pre-publication history

The pre-publication history for this paper can be accessed here:


